# Single LED, Single PD-Based Adaptive Bayesian Tracking Method

**DOI:** 10.3390/s22176488

**Published:** 2022-08-29

**Authors:** Duckyong Kim, Jong Kang Park, Jong Tae Kim

**Affiliations:** Department of Electrical and Computer Engineering, Sungkyunkwan University, Suwon 16419, Korea

**Keywords:** Bayesian methods, indoor positioning system (IPS), sensor tracking, sensor fusion, visible light positioning (VLP)

## Abstract

Recently, with the growing interest in indoor location-based services, visible light positioning (VLP) systems have been extensively studied owing to their advantages of low cost, high energy efficiency, and no electromagnetic interference. However, due to structural limitations which lead to the requirement of multiple signal sources, it has been challenging to apply VLP in real-world scenarios. In this study, we propose a single LED, single PD-based tracking system that solves these problems by applying a new Bayesian method that can effectively reduce the computational burden of particle filters. The method of evaluating particle reliability developed in this work adjusts the number of particles on the fly. Using the absolute position of the single LED source, the long-term cumulative error of the inertial measurement unit can be continuously corrected. In this regard, the applicability of the VLP system can be enhanced in places where the multiple luminescent signals are hard to consistently detect. The proposed system was verified through experiments in a classroom and a corridor, and the results show an average error of less than 11 cm at travel distances of 80 to 100 m.

## 1. Introduction

In recent years, extensive research has been conducted on indoor positioning, where satellite signals are typically interrupted and degraded. Indoor positioning systems (IPSs) are configured through various types of signals, such as infrared and Wi-Fi, which have problems including short transmission distances, multipath effects, low accuracy, various types of signal interference, and the high cost of building such systems. To overcome these challenges, several studies have been conducted on visible light communication (VLC)-based positioning systems. By using light-emitting diodes (LEDs) as signal sources, VLC-based systems feature energy efficiency, low cost, long lifetime, and significant advantages in terms of construction costs because they use the LED infrastructure already built into the use environment [1,2,3,4]. In VLP systems, the most preferred method for low cost and high performance systems is the photodiode (PD)-based intensity-modulation/direct-detection (IM/DD) VLP system.

Because existing optical positioning systems are based on trilateration, at least three pieces of LED-to-PD signal information are essential [1,5]. This fundamental structure causes VLP systems to have the following limitations in real-life applications.

**Figure 1:**
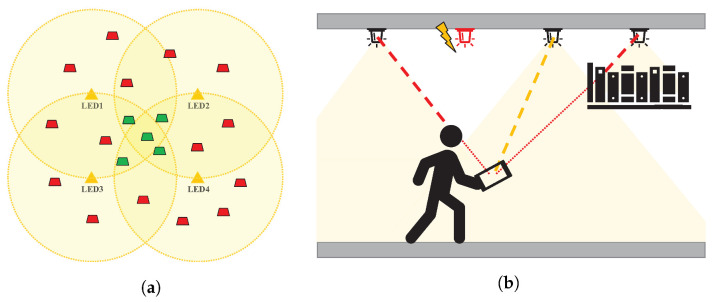
Structural problem of general multiple signal-based VLP systems: (**a**) problems caused by restrictions on viewing angles and (**b**) problems caused by obstacles, failures, etc.

- All LED signals must satisfy the line-of-sight (LOS) condition based on the element’s field of view (FOV) to receive multiple LED-PD signal information [1].
  - In Figure 1a, the area in which an optical signal can be stably received from each LED is fairly large (yellow circles ), while the area in which multiple signal-based positioning can be performed is quite limited (green trapezoids). Even if one or more signals can be stably received in the remaining area, positioning them is structurally impossible (red trapezoids).
  - LOS may not be guaranteed in various indoor and outdoor environments due to unexpected obstacles, user shadows, and extreme situations such as element failure (Figure 1b). If positioning is performed based on a single optical signal, the resulting area of positioning can be very wide.
- When the radiation and incident angles exceed the FOV of each element, the error in the received signal gain increases exponentially [5]. Even if the FOV is adjusted to handle this problem, a large FOV results in poor accuracy owing to ambient or reflective light [6], whereas a small FOV results in the PD not receiving LED signals correctly [7].
- There can be issues with LED management and reliability. Depending on the device characteristics, the junction temperature of an LED may change due to the driving current, ambient temperature, and self-heating. The main wavelength, signal efficiency, and characteristics of the LED may change according to changes in temperature [8,9,10]. LED signal power may degrade over time, creating error directionality and adversely affecting overall positioning accuracy [1,11].
- Additionally, the use of multiple LED channels induces interference effects such as crosstalk between channels [12,13,14] and inter-cell interference (ICI) [15,16,17].
- Multiple PD-based VLP methods [18,19,20,21,22,23] have been developed to produce multiple LED-to-PD signals in a single-LED environment. These studies include a method using a horizontal PD arrangement spaced at arbitrary intervals [18,19], a method using angular diversity detectors [20,21], and a method combining the two methods [22,23]. However, these methods require bulky receiving devices, and it is difficult to secure the viewing angle for all PDs.
- There exist a few recent studies exploiting a single LED signal. Image sensor arrays can capture the geometrical features of a single LED signal for complete identification [24,25]. However, such devices typically have limited scan rates (30–60 Hz) for the entire sensor array. It is difficult to increase the modulation frequency and extend signal bandwidths. This further limits the noise immunity of VLP systems. Li et al. employed a special LED lamp for VLP, although this required additional small luminescent beacons [26].
- The general positioning environment, where the receiver and transmitter are not facing each other in parallel and the height is not limited, has not been sufficiently studied [2,6,27]. Because the optical signal pattern includes the cosine of the incidence angle (ψ) and the radiation angle (ϕ), the calculation becomes very complicated when the receiver is not horizontal. The effect of the *X*- and *Y*-axis movements is greater than the effect of the *Z*-axis movement, and it is relatively difficult to accurately estimate the height of the receiver. In our previous study [2], we investigated the existing problems with conventional VLP studies and analyzed in detail the reasons that these problems were difficult to solve. However, in [28], a new statistical model for device orientation was established based on the observation results of several participants using a smartphone. The authors showed that an average polar angle of $30^{\circ }$ with a standard deviation of $9^{\circ }$ occurred during walking activities.

In general, inertial measurement units (IMUs) are among the most common elements found in electronic devices, and are mainly used to estimate the posture of the device. The inertial navigation system (INS) is a dead-reckoning navigation system that acquires dynamic information through direct measurement from an IMU sensor. INS is the most basic method of sensor tracking systems due to its simple implementation. However, because INS estimates the current position from the previous position with current measurements, there is a fatal problem when the starting position is unknown or the system is unexpectedly restarted. In addition, inertial measurement devices are vulnerable to drift and environmental errors in which the reference output value of the sensor is affected in a static state [29]. Although great performance can be shown in the short-term, the long-term error accumulates as the driving time increases by taking the form of integration [30].

In this study, we propose a single-LED, single-PD-based sensor tracking algorithm using an optical sensor and an IMU sensor. To the best of our knowledge, a method for three-dimensional (3D) position estimation exploiting a single optical signal has not been presented in any other study to date. The proposed method is a light-based adaptive particle filter (Li-APF) developed using a novel Bayesian tracking technique. A particle filter (PF) is a trial and error-based prediction algorithm that is highly effective for estimating the state of nonlinear systems with non-Gaussian noise distributions based on probability density distributions. However, the fundamental problem for precise positioning is that the number of calculations increases as the number of particles increases. The proposed method effectively reduces the computational cost of particle filters by utilizing optical signal characteristics. By combining particle positioning and INS, we implemented a complete tracking system that compensates for the drawbacks of each sensor. The characteristics of the proposed method are summarized as follows:

1. By requiring only one pair of optical signals, most of the fatal problems of conventional multi-source VLPs are solved. Because the proposed method requires the stable reception of only one signal, the operational coverage is very wide (Figure 1a, red trapezoids) and there are few restrictions due to obstacles (Figure 1b). Furthermore, by combining the INS results, positioning can be maintained even in a region where optical signals are blocked.
2. The proposed method can effectively adjust the initial distribution of the particle filter using the optical signal intensity information. We propose an index called “particle reliability” which evaluates the overall particle behavior. Through this index, the number of particles is flexibly adjusted; thus, the calculation burden of the particle filter is reduced. Online tracking is guaranteed for a nonlinear system with non-Gaussian noise by effectively reducing the computational cost of the particle filter.
3. Based on the absolute position of the LED signal, the system itself can estimate its own position even if it does not have any previous state information. The proposed algorithm corrects the long-term cumulative error of the INS based on the continuity of optical positioning.

The remainder of this paper is organized as follows. In Section 2, we describe the optical model used in the particle calculation process. Section 3 describes the proposed tracking algorithm. The overall flow is introduced and explained in detail for each part. In Section 4, we verify the particle update process, which is the core of the proposed algorithm, through simulation. In Section 5, we describe an indoor tracking experiment with the proposed method using a field-programmable gate array (FPGA) and simple hardware, then discuss its numerical results. Finally, we present our conclusions in Section 6.

## 2. Optical Model Representation in Cartesian Coordinate System

**Figure 2:**
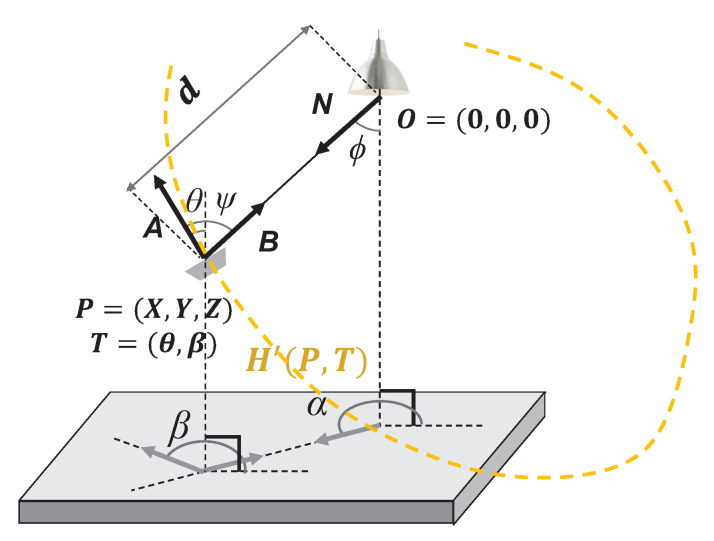
Optical system representation in 3D space.

The proposed algorithm uses the ideal optical signal strength value according to the position and tilt of each particle in the initialization and particle updating processes. Figure 2 shows an optical signal transmission system. Assuming that the position of the LED is $L_{O}\left(x_{L},y_{L},z_{L}\right)$ and the position of the device is $X_{O}\left(x_{X},y_{X},z_{X}\right)$, the corrected position of the device assuming the LED as the origin is $P\left(X,Y,Z\right)=\left(x_{X}-x_{L},y_{X}-y_{L},z_{X}-z_{L}\right)$. The horizontal and vertical tilt of the PD is represented by $T\left(\beta ,\theta \right)$, and α is the horizontal angle at which the transmitter points toward the receiver, while ϕ and ψ are the radiation and incident angles with respect to the transmitter and receiver, respectively. When the unit vector of the receiver tilt and the direction of the device and the LED facing each other are A, B, and N, respectively, the incident angle ψ is expressed by the inner product of the two vectors as follows:

(1) $$\begin{array}{c} \cos\psi =\frac{A\cdot B}{\left|A\right|\left|B\right|}=\cos\theta \cos\phi \;-\;\sin\theta \sin\phi \cos\left(\beta \;-\;\alpha \right) \end{array}$$

In addition, as $P\left(X,Y,Z\right)=d\times N,N=\left(\sin\phi \cos\alpha ,\sin\phi \sin\alpha ,\cos\phi \right)$, the horizontal and vertical angles of the spherical coordinate system are expressed as follows:

(2) $$\begin{array}{c} \cos\phi =\frac{Z}{\sqrt{X^{2}+Y^{2}+Z^{2}}},\;\;\sin\phi =\frac{\sqrt{X^{2}+Y^{2}}}{\sqrt{X^{2}+Y^{2}+Z^{2}}},\;\;\cos\alpha =\frac{X}{\sqrt{X^{2}+Y^{2}}},\;\;\sin\alpha =\frac{Y}{\sqrt{X^{2}+Y^{2}}} \end{array}$$

Here, by applying the above to the Lambertian radiation pattern [31], the ideal optical signal strength $H^{\prime }$ received from the device is derived as follows:

(3) $$\begin{array}{cc} H^{\prime }\left(P,T\right) & =\frac{(m+1)A}{2\pi }\cdot \cos^{m}\phi \cos^{n}\psi \cdot d^{-2}\\ & =K\;\cdot \;Z^{m}\;{\left(\cos\theta Z\;-\;\sin\theta \cos\beta X\;-\;\sin\theta \sin\beta Y\right)}^{n}{\left(X^{2}\;+\;Y^{2}\;+\;Z^{2}\right)}^{\left(-2-m-n\right)/2}, \end{array}$$

where *A* is the physical area of the light detector and *d* is the distance between the LED and PD. In addition, $m=\frac{-\ln2}{\ln\left(\cos\left(\phi_{1/2}\right)\right)}$ and $n=\frac{-\ln2}{\ln\left(\cos\left(\psi_{1/2}\right)\right)}$, where $\phi_{1/2}$ and $\psi_{1/2}$ are the half angles of the transmitter and receiver, respectively. By converting the signal strength model from a spherical coordinate system to a Cartesian coordinate system, which is a major factor in the particle update process, the calculation of signal strength can be simplified.

## 3. Single Visible Light Signal-Based Tracking Algorithm

In this study, we propose a new Bayesian tracking method, Li-APF. The proposed method can effectively reduce the computational cost of the particle filter by requiring a very small number of particles and managing them efficiently. Using the results of particle estimation and the INS, we compensate for the drawbacks of each result prior to the final decision. The proposed method uses the dead reckoning principle with inertial sensor data. By additionally referring to the absolute location information of the LED and the distance of the received signal strength (RSS), it is possible to operate without any initial information. Additionally, the INS error is compensated for by continuous positioning using the optical signal information.

**Figure 3:**
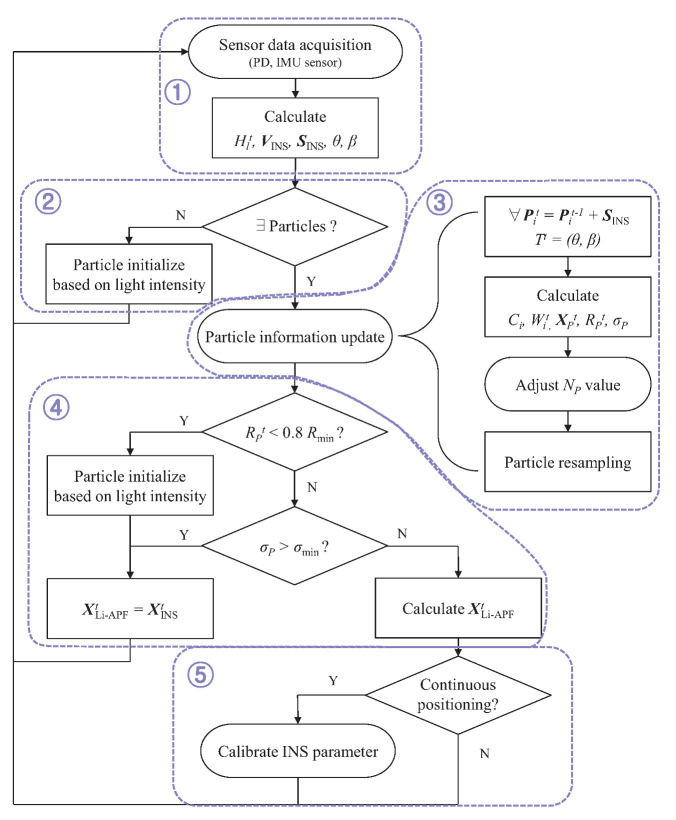
Overall flowchart of Li-APF algorithm.

Figure 3 shows an overall flowchart of the proposed tracking algorithm. The algorithm is divided into five steps. In the first step (①), raw sensor data are measured using an optical sensor and an IMU sensor, then the data are converted for use in an algorithm. Next, if the initial positioning is performed without any particle information, particle initialization (②) is performed; otherwise, the particle update process (③) is performed. The particle update process is the core process of the proposed algorithm, and includes particle unit movement, weight updating through the cost function, particle positioning, and resampling. The proposed method reduces the direct computational burden by efficiently reducing the total number of particles and further simplifies the calculation equation by modifying the optical signal intensity model to facilitate positioning analysis. In the next step (④), the particle positioning result is evaluated and the final position is determined according to the evaluation results. Finally, the INS error-correcting process (⑤) is conditionally added. Each of these five processes is described in detail in the following subsections. The primary parameters are listed in Table 1.

### 3.1. Sensor Data Acquisition and Analysis Process

First, environmental data are collected through PD and IMU sensors and the values are processed for use in the positioning algorithms. In the case of IM/DD-based optical signals, signals from various channels are combined and received; in general, the signal strength $H_{l}^{t}$ for each channel is classified by applying multiplexing and demultiplexing. The reference signal ($H^{t}$) that determines the device position was selected for the largest received optical signal intensity ($H_{l}^{t}$). The IMU sensor data output the local data, that is, the data based on the sensor orientation, meaning that it should be converted into global coordinates. To this end, the tilt of the sensor (θ,β) is first estimated through the Attitude and Heading Reference System (AHRS) based on the sensor data, then the sensor data are converted into global coordinates by reverse correction of the posture. In addition, the movement speed $V_{\mathrm{INS}}$ and unit time movement distance $S_{\mathrm{INS}}$ of the current device are calculated through the accumulation of acceleration data.

### 3.2. Particle Initialization Process

**Figure 4:**
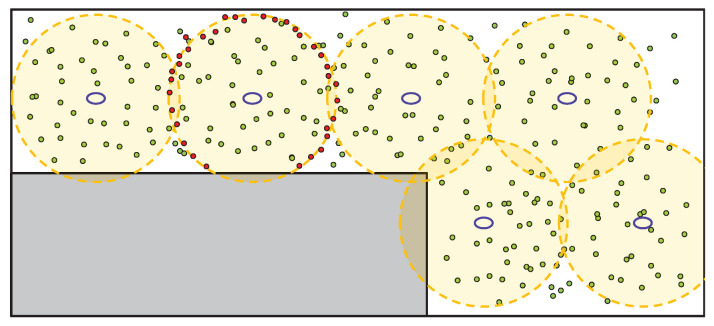
Example of particle initialization (green dot: general PF; red dot: Li-APF).

After analyzing the sensor data, the next step is to check whether there are previously generated particles, and if not, to initialize the particles. Examples of particle initialization with both the general PF method and the proposed method are shown in Figure 4. The purple ovals in the figure indicate the positions of the LEDs. In the proposed system, the ideal optical signal strength can be calculated based on the distance from the LED, meaning that the initial random particle is set for the area that meets the signal strength condition (red dot), not for the entire map area (green dot). This condition effectively limits the initial particles. The initial position of particle $P_{i}^{0}$ is set to satisfy the following condition:

(4) $$\begin{array}{c} \left\{\begin{array}{c} P_{i}^{0}=\left(r_{i}\cdot \cos\left(2\pi i/N_{P}\right),\;r_{i}\cdot \sin\left(2\pi i/N_{P}\right),h_{i}\right)\;\\ \left|H^{\prime }\left(P_{i}^{0},T^{0}\right)-H^{0}\right|<\epsilon_{H} \end{array}\right. \end{array}$$

where $r_{i}$ and $h_{i}$ are the distance between the LED and the particle in the x–y plane and z plane, respectively. Each particle has state information $\left(P_{i}^{0},T^{0}\right)$ and weight values. In the initial stage, the weights of all the particles are initialized to the same $W_{\mathrm{init}}$ value. Because there is no connection with the previous time, the particle update process cannot be performed, and the process continues to the next acquisition.

### 3.3. Particle Updating Process

If previously generated particles exist, the particle updating process is performed. In this process, various probability particles distributed in space are virtually moved using the unit time movement information from the IMU sensor data. The probability weight of each particle is updated using the cost function calculated by comparing the ideal value with the measured value of the previous and current optical signal strengths. Thereafter, the weighted average of the updated particles is determined as the current position, and particles with low weight are discarded and regenerated randomly.

When the previous and current states of the *i*-th particle are $\left(P_{i}^{t-1},T^{t-1}\right)$ and $\left(P_{i}^{t},\;\;T^{t}\right)$, they can be derived as $P_{i}^{t}=P_{i}^{t-1}+S_{\mathrm{INS}}$. After moving all particles, the cost function for each particle is calculated. The desired value determined through the cost function is the degree of the positional error of the positioning target, which is related to the distance. Because the distance *d* is a value proportional to the −1/2 power of the signal strength, the ratio is calculated by matching the measured signal strength and the ideal signal strength proposed in this study to the distance level. In addition, because the probability of the current particle and the probability of the previous particle must be considered together in order to derive the exact cost of the particle moving at a continuous measurement, it is expressed in one equation by multiplying the signal intensity ratio of the two values. Then, the square root calculation is performed because the whole expression is again expressed as a value of the square level of the distance, and its reciprocal makes a smaller error have a larger. Here, the cost function value $C_{i}$ of the particle is calculated as follows:

(5) $$\begin{array}{c} C_{i}\;=1/\;\;\sqrt{\left|\frac{\sqrt{H^{\prime }\left(P_{i}^{t-1}\;,T^{t-1}\right)}}{\sqrt{H^{t-1}}}\;-\;1\right|\;\cdot \;\left|\frac{\sqrt{H^{\prime }\left(P_{i}^{t}\;,T^{t}\right)}}{\sqrt{H^{t}}}\;-\;1\right|} \end{array}$$

After calculating the cost function of each particle, the weight is updated. The weight is calculated as the sum of the previous weight and the current cost function at a constant ratio, $r_{W}:1-r_{W}$ (Equation (6)). Here, if the sampling frequency is $f_{s}$ and the stabilization time is $T_{\mathrm{stable}}$, the weight update rate $r_{W}$ is adjusted according to Equation (7). The stabilization time is arbitrarily designated by the user, and refers to the time required for the measurement of the accumulated data to affect the result by more than 90%:

(6) $$\begin{array}{cc} & W_{i}^{t}=W_{i}^{t-1}\cdot r_{W}+C_{i}\cdot \left(1-r_{W}\right)=W_{\mathrm{init}}\cdot {\left(r_{W}\right)}^{t}+\cdots +C_{i}\cdot \left(1-r_{W}\right) \end{array}$$

(7) $$\begin{array}{ll} & {\left(r_{W}\right)}^{T_{\mathrm{stable}}\cdot f_{s}}<0.1\;\Leftrightarrow \;∴r_{W}<0.1^{1/(T_{\mathrm{stable}}\cdot f_{s})} \end{array}$$

In this study, values of $f_{s}$ = 10 Hz, $T_{\mathrm{stable}}$ = 0.5 s, and $r_{W}\approx$ 0.631 were applied.

After updating the weight of all particles, the result of the particle positioning $X_{P}^{t}$ is calculated by obtaining the weighted average value of all particles based on the updated weights. However, particles are scattered in various directions with respect to the LED. When all particles are considered, the average coordinate is inevitably designated as a location close to the LED. Therefore, the final position is calculated by considering only the upper 50% weights of the particles with the highest values. In addition, the absolute position compensates for the adjusted relative position based on the LED position. When the set of particles with the upper 50% weights is U, the estimated position $X_{P}^{t}$ is derived as follows:

(8) $$\begin{array}{c} X_{P}^{t}=W_{i\in U}^{t*}\times P_{i\in U}^{t}+L_{O}, \end{array}$$

where $W_{i\in \;U}^{t*}$ represents a normalized weight of upper 50% particles.

Next, the particle reliability $R_{P}^{t}$ is calculated using the weighted average of the cost function based on the updated weights of all particles and the currently received signal strength, as follows:

(9) $$\begin{array}{c} R_{P}^{t}=\sum_{i}^{N_{P}}W_{i}^{t*}C_{i}\cdot \sqrt{H^{t}}, \end{array}$$

where $W_{i}^{t*}$ represents the normalized weight of all the particles. By taking the weighted average, the index of reliability can be defined using the average cost function and the degree of positioning error accordingly, further considering that the signal strength becomes smaller as the distance from the LED increases. Because the reliability is based on the average cost function, it is considered to be an indicator of whether the overall behavior of particles matches the measured signal information.

In this study, the expected standard reliability $R_{\mathrm{typ}}$ and minimum reliability $R_{\min}$ are explicitly defined, allowing the current particle reliability $R_{p}^{t}$ to be evaluated during the Li-APF procedure. These parameters are used to determine the particle numbers and the resulting position from INS and PF estimates. When the arbitrary representative reference coordinates of the previous and current position are $P_{\mathrm{typ}}^{t-1},P_{\mathrm{typ}}^{t}$ and the actual device location is $P_{O}^{t-1},P_{O}^{t}$, the ideal values of the measured signal intensity are expressed as $H^{t-1}=H^{\prime }\left(P_{O}^{t-1},T^{t-1}\right)$ and $H^{t}=H^{\prime }\left(P_{O}^{t},T^{t}\right)$. If the distance calculated by any $P_{k}$ is $D_{k}={\left(X_{k}^{2}+Y_{k}^{2}+Z_{k}^{2}\right)}^{1/2}$, the representative cost function can be expressed as (10). This equation is constructed based on (5) mentioned above.

(10) $$\begin{array}{c} C_{\mathrm{typ}}=1/\sqrt{\;\left|\frac{\sqrt{H^{\prime }\left(P_{\mathrm{typ}}^{t\;-\;1},T^{t\;-\;1}\right)}}{\sqrt{H^{\prime }\left(P_{O}^{t\;-\;1},T^{t\;-\;1}\right)}}\;-\;1\right|\;\cdot \;\left|\;\frac{\sqrt{H^{\prime }\left(P_{\mathrm{typ}}^{t},T^{t}\right)}}{\sqrt{H^{\prime }\left(P_{O}^{t},T^{t}\right)}}\;-\;1\right|}=1/\sqrt{\left|\frac{D_{\mathrm{typ}}^{t-1}-D_{O}^{t-1}}{D_{O}^{t-1}}\right|\cdot \left|\frac{D_{\mathrm{typ}}^{t}-D_{O}^{t}}{D_{O}^{t}}\right|} \end{array}$$

Here, if the difference between the representative coordinates and the actual coordinates, that is, the error distance, is $\Delta_{\mathrm{typ}}^{t-1}=D_{O}^{t-1}-D_{\mathrm{typ}}^{t-1},\Delta_{\mathrm{typ}}^{t}=D_{O}^{t}-D_{\mathrm{typ}}^{t}$, and $\Delta_{\mathrm{typ}}^{t-1}\approx \Delta_{\mathrm{typ}}^{t}=\Delta_{\mathrm{typ}},D_{O}^{t-1}\approx D_{O}^{t}=D_{O}$, then $C_{\mathrm{typ}}$ can be further simplified as below. As the sampling times of sensor tracking systems are short enough, this assumption is convincing.

(11) $$\begin{array}{c} C_{\mathrm{typ}}=\sqrt{\frac{D_{O}^{t-1}\cdot D_{O}^{t}}{\Delta_{\mathrm{typ}}^{t-1}\cdot \Delta_{\mathrm{typ}}^{t}}}\approx \frac{D_{O}}{\Delta_{\mathrm{typ}}} \end{array}$$

By setting the reference conditions as $\phi =\phi_{1/2}$ and $\psi =\psi_{1/2}$ and applying this to Equation (9), the standard reliability can be derived as follows:

(12) $$\begin{array}{cc} R_{\mathrm{typ}} & =C_{\mathrm{typ}}\cdot \sqrt{H^{\prime }\left(P_{O}^{t},T^{t}\right)}\approx \sqrt{K\cdot \cos^{m}\phi_{1/2}\cos^{n}\psi_{1/2}}\cdot \frac{1}{\Delta_{\mathrm{typ}}}=\frac{\sqrt{K}}{2}\cdot \frac{1}{\Delta_{\mathrm{typ}}}. \end{array}$$

This equation shows that $R_{\mathrm{typ}}$ is inversely proportional to the reference error that the user can arbitrarily designate. Similar to the representative reliability, the minimum reliability is derived as follows:

(13) $$\begin{array}{c} R_{\min}\approx \frac{\sqrt{K}}{2}\cdot \frac{1}{\Delta_{\min}}. \end{array}$$

In this study, we apply $\Delta_{\mathrm{typ}}$ = 0.05 m and $\Delta_{\min}$ = 0.5 m for $R_{\mathrm{typ}}$ and $R_{\min}$, respectively.

Next, we calculate the weighted standard deviation of the particle angle $\sigma_{P}$, which can determine the degree of distribution of all particles. Unlike particle reliability, this value is an indicator that evaluates whether the estimated position $X_{P}^{t}$ as a weighted average is sufficiently reliable. To calculate the degree of spreading of particles in an elliptical shape based on the LED, the angle between the estimated position and each particle, $\omega_{i}$, is calculated around the LED. The weighted standard deviation of the spread angle is then calculated.

(14) $$\begin{array}{cc} & \omega_{i}=\mathrm{atan}\left(X_{P}^{t}\right)-\mathrm{atan}\left(P_{i}^{t}\right) \end{array}$$

(15) $$\begin{array}{cc} & \sigma_{P}=\sqrt{\sum_{i}^{N}W_{i}^{t*}{\left(\omega_{i}-\bar{\omega_{i}}\right)}^{2}} \end{array}$$

Here, atan(·) is a function representing an arctangent value on the x–y coordinate of the parameter vector based on the *x*-axis. In this study, we applied the minimum angle deviation as $\sigma_{\min}=45^{\circ }$.

Prior to particle resampling, the amount of computation can be efficiently reduced by adjusting the total number of particles, depending on the situation. The particle positioning performance can be classified into three sections based on the particle reliability value, namely, excellent, good, and bad. We set the minimum particle number that can guarantee accurate positioning when the particle performance is excellent. Conversely, we check the maximum number that allows the particle distribution to converge correctly within a certain time while the performance is bad. This study was designed to adjust the number of particles $N_{P}$ according to the following criteria:

(16) $$\begin{array}{c} N_{P}=\left\{\begin{array}{c} 10,\;\;when\;\;\;\;\;\;\;\;R_{P}^{t}>0.8\;R_{\mathrm{typ}}\\ 30,\;\;when\;\;\;\;\;\;0.8\;R_{\mathrm{typ}}\geq R_{P}^{t}>1.2\;R_{\min}\;\\ 50,\;\;when\;\;\;\;1.2\;R_{\min}\;\geq R_{P}^{t} \end{array}\right.\;\;\;\;\;\; \end{array}$$

Through this process, if the number of particles decreases, the particles with the lowest weight are removed by that number, and if the number increases, an additional particle with $W_{i}^{t}=0$ is generated. The position of the new particles is determined through the resampling process.

Particle resampling is the final step in the particle updating process, in which particles with low weights are discarded and new particles are created at random candidate locations. In this study, particles with $W_{i}^{t}<0.5\;W_{\mathrm{init}}$ are rearranged to satisfy the following optical signal strength condition:

(17) $$\begin{array}{c} \left\{\begin{array}{c} P_{i}^{t}=\left(r\cdot \cos(2\pi \cdot RN),r\cdot \sin(2\pi \cdot RN),h\right)\;\\ \left|H^{\prime }\left(P_{i}^{t},T^{t}\right)-H^{t}\right|<E_{H} \end{array}\right.\;\;\;\;\;, \end{array}$$

where RN is any random number between zero and one. The weights of all resampled particles are initialized to $W_{\mathrm{init}}$ value.

### 3.4. Decision through Weighted Average with INS

The aforementioned particle updating process is considered to be based on the optical signal intensity information. However, when the reliability of optical signal reception is degraded due to defects in LED and PD elements or temporary signal distortion, it directly affects the entire system. To address this issue, we use an integrated INS tracking system. However, because the cumulative error is significant in the INS system, in this study the INS positioning result is determined by applying the current unit time movement from the previous integrated result, as follows:

(18) $$\begin{array}{c} X_{\mathrm{INS}}^{t}=X_{\mathrm{Li}-\mathrm{APF}}^{t-1}+S_{\mathrm{INS}} \end{array}$$

By deriving the positioning results of the particle filter and INS in each process and weighting them according to the ratio $R_{P}^{t}/R_{\mathrm{typ}}:1$, the final estimated position $X_{\mathrm{Li}-\mathrm{APF}}^{t}$ is determined as follows:

(19) $$\begin{array}{c} X_{\mathrm{Li}-\mathrm{APF}}^{t}=\frac{R_{P}^{t}\cdot X_{P}^{t}\;+\;R_{\mathrm{typ}}\cdot X_{\mathrm{INS}}^{t}}{R_{P}^{t}\;+\;R_{\mathrm{typ}}}. \end{array}$$

**Figure 5:**
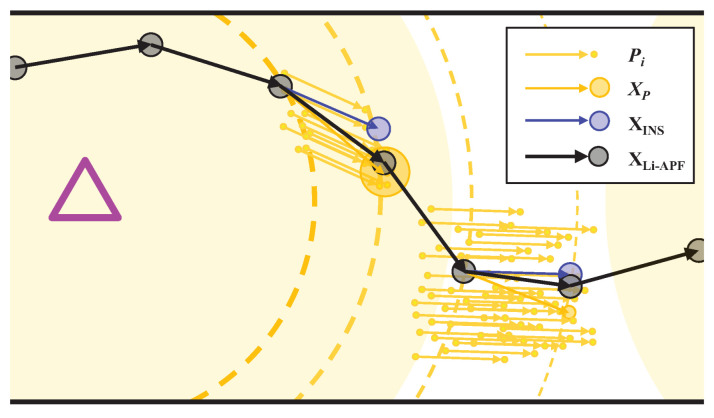
Brief representation of the process of the proposed algorithm.

Figure 5 shows a brief portion of the operation of the proposed tracking system. In the figure, the purple triangle indicates the location of the LED. This expression is commonly applied to the following figures. The particle movement of the Li-APF and the current estimated state (particle-weighted mean) are shown as yellow lines and circles, and its reliability is represented as the size of the circle. The estimated states of the INS and weighted average decision are shown as blue and black circles. In the third estimation stage in the figure, because the received LED signal is sufficiently stable, the reliability of Li-APF is high, and the weighted average of the final estimation is focused on the Li-APF result. Additionally, owing to the high reliability, the total number of particles in the previous step decreases. Conversely, in the fifth estimation, the LED signal is relatively unstable, meaning that the reliability of Li-APF decreases, the number of particles increases, and the final judgment is biased on the INS result.

### 3.5. INS Calibration Using Particle Information

**Figure 6:**
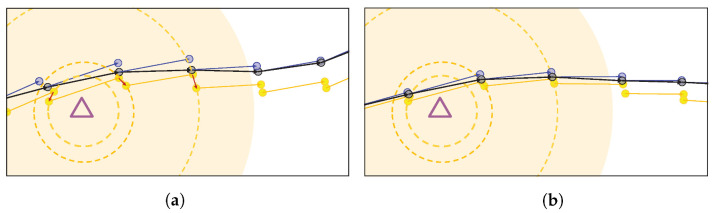
Example of algorithm operation according to INS error: (**a**) when INS accumulated error occurs and (**b**) when the accumulated INS error is corrected.

After estimating the current device’s position through the aforementioned processes, an additional process of calibrating the accumulated error of the INS is performed using particle information based on the optical signal. Figure 6 shows a problem that may arise due to the accumulation of INS errors, that is, the cumulative speed error. In this figure, it can be observed that the estimated result (black circle) is continuously derived according to the weighted average of the particle position (yellow circle) and the INS position (blue circle). In Figure 6a, which is largely influenced by the cumulative error of the INS, it can be seen that depending on the cost function, particle positioning operates in a standard manner for optical signal strength conditions, while the continuity of previous and current particles is not smooth enough (red arrow) due to the long-term error of INS. If this aspect continues, the reliability and deviation of the particles deteriorates and the error of the INS increases, resulting in fatal consequences for the overall system. Likewise, as the device moves away from the LED and the optical signal intensity weakens, the optical reliability decreases and the influence of the INS on the final positioning increases, leading to the same result. Figure 6b shows the state in which the INS error is corrected and the connection between the previous and current particles is improved. Even when the optical signal strength is weakened, this can guarantee overall positioning performance because the INS error has been corrected.

In this study, the difference between the consecutive points of the particles is used to compensate for this unit time movement error. For this, the previous particle positioning results and reliability should be stored in $X_{P}^{t-1}$ and $R_{P}^{t-1}$. Then, the INS variable can be adjusted using the previous and current particle filter values, as follows:

(20) $$V_{\mathrm{INS}}=V_{\mathrm{INS}}+\lambda_{\mathrm{INS}}\cdot \left(\left(X_{P}^{t}-S_{\mathrm{INS}}\right)-X_{P}^{t-1}\right)$$

(21) $$\lambda_{\mathrm{INS}}=\lambda \;\left(R_{P}^{t-1}\cdot R_{P}^{t}/R_{\mathrm{typ}}^{2}\right)$$

Here, the adjusting value is directly updated to $V_{\mathrm{INS}}$ before being accumulated in the next data collection and analysis process. In this study, the acceleration correction ratio λ is applied as 0.1 s^−1^.

However, as can be seen from the flowchart in Figure 3, INS error correction uses the difference between the previous and current particle positioning results; thus, this process cannot be used when the reliability or distribution of the particle is poor.

## 4. Particle Update Simulation

In this section, we verify the functionality of the proposed algorithm in an environment where initial positional information is not specified. In this simulation, we focus on particle update behavior, not the entire process of the proposed algorithm. Therefore, this simulation result does not represent the final result of the proposed algorithm; the entire algorithm is practically evaluated in Section 5. We observe the movement of each particle and the convergence performance to the solution, and check the $R_{P}^{t},\sigma_{P}$, and $X_{\mathrm{Li}-\mathrm{APF}}^{t}$ values. The simulation is configured as follows. A single LED is located at a position (0 m, 0 m, 0 m) directly facing the floor. The device moves around the LED within a radius of 2 m, changing the vertical tilt randomly from $-30^{\circ }$ to $+30^{\circ }$ with a speed of approximately 1 m/s. The height of the device is designated as 1.5 m. We measure the sensor data at a rate of 10 Hz for 3 s. In this simulation, an optical channel model with a signal-to-noise ratio of 30 dB is assumed and the noise from the IMU sensor is not considered. The representative error and the minimum error are set to $\Delta_{\mathrm{typ}}$ = 0.05 m and $\Delta_{\min}$ = 0.5 m, and thus the representative and minimum reliability values are $R_{\mathrm{typ}}=27.9\times 10^{6},R_{\min}=278.7\times 10^{6}$. The reference deviation is set to $\sigma_{\min}=45^{\circ }$.

**Figure 7:**
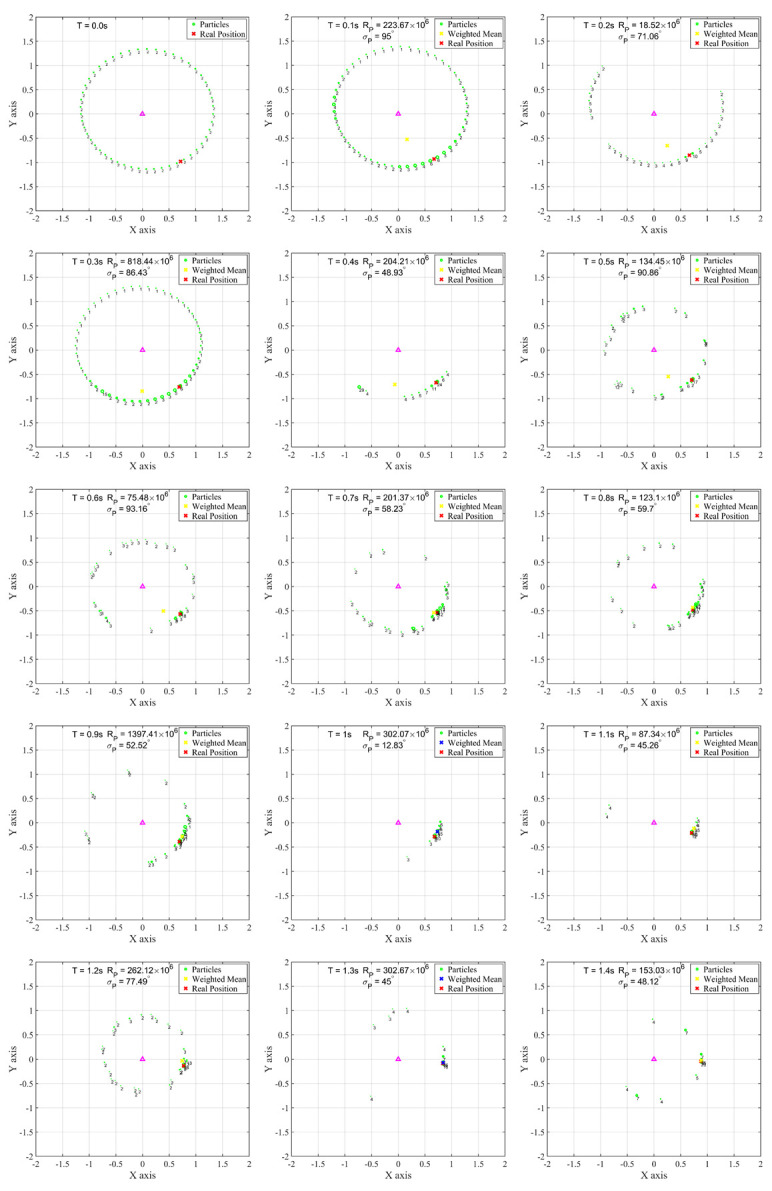
Timeline representation of the particle update process.

Figure 7 shows the particle updating process for each sampling time. In this figure and the following figure, the purple triangle indicates the location of a single LED. The green circle represents the position of each particle, and the size of the circle represents the cost function value of each particle proportionally. The numbers at the bottom of each particle represent integer digits of the percentage used for normalizing the updated weights of the entire particle. The red X represents the actual position of the moving device during the simulation. The X marked in yellow or blue is the position of the device calculated using the current particle information ($X_{P}^{t}$). Under the conditions of weighted deviation, if the particle positioning is judged to be valid with a small $\sigma_{P}\;\left(\leq \sigma_{\min}\right)$, it is marked in blue, and if it is not valid ($\sigma_{P}>\sigma_{\min}$), it is marked in yellow. In the actual positioning process, invalid yellow X information is excluded. The current time *T*, particle reliability $R_{P}^{t}$, and weighted deviation $\sigma_{P}$ values are indicated at the top of each sample. In the initial state, fifty particles are uniformly spread to ideal device candidate positions according to the first received optical signal strength, all of which have weight values of the same size. Because there is no cost function calculation, the size of the circle is adjusted to have the same value. In addition, it can be observed that the initial particle distribution has an elliptical trajectory due to the tilt of the sensor ($\theta =29.32^{\circ },\beta =230.45^{\circ }$). After 0 s, the particle updating process begins. The cost function is large in particles near the actual device location, although particles in other locations show large cost functions as well. However, this pattern disappears after several particle updates. In addition, the number of particles is adjusted to thirty after 0.1 s, as the particle reliability $R_{P}^{t}$ is greater than $1.2R_{\min}$. At 0.3 s and 0.9 s, the number of particles is adjusted to ten according to the $R_{P}>0.8R_{\mathrm{typ}}$ condition. Meanwhile, the value of each weighted deviation increases as the particles are evenly spread, and the deviation decreases as the particles become concentrated. In Figure 7, it can be seen that the distribution of particles after 1 s is relatively stable and there is little positioning error.

**Figure 8:**
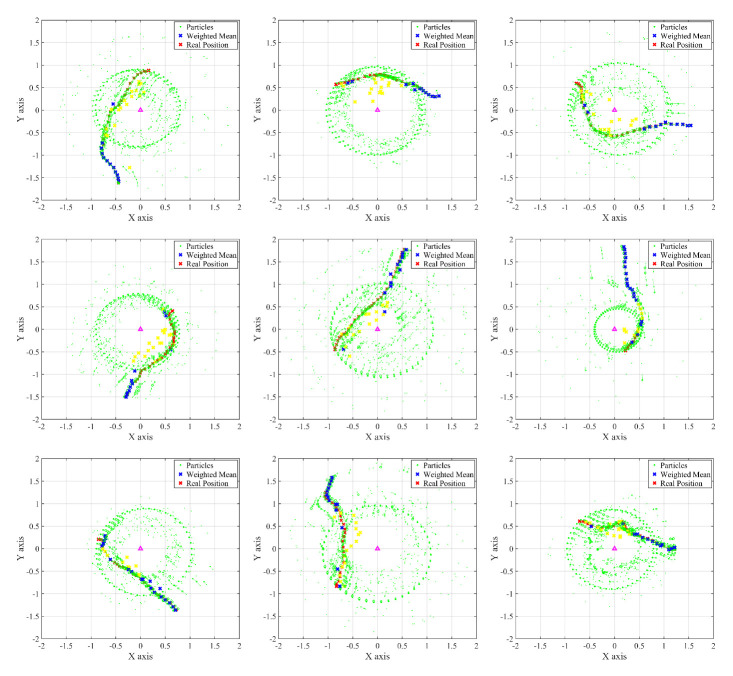
Cumulative representation of particle updates in random trials.

Figure 8 shows the particle initialization and position-determining process in various random environments. The particle updates for one simulation are accumulated and shown in each subfigure. In addition, for particles with information that is continuously updated without being removed at the previous time, a green line shows this continuity by connecting their previous and current positions. As shown in this figure, the actual position of the device can be accurately estimated within 1 s to at least 2 s using the proposed particle filter. It can be observed that the stable positioning result after 2 s has almost no error as compared the actual device position.

## 5. Experiment

**Figure 9:**
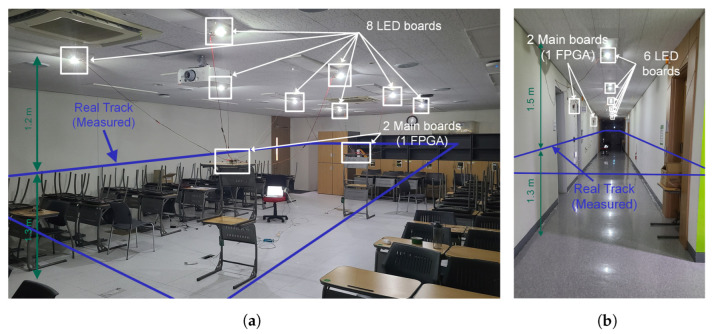
Experimental environment: (**a**) classroom (eight LEDs) and (**b**) corridor (six LEDs).

In this section, we evaluate the tracking accuracy of the proposed algorithm through experiments in indoor environments. Although several LEDs can be used for experiments, our system consists of a single LED-based method that uses only the single strongest signal for each sample. In the experiments described here, the proposed system was validated in indoor environments with the user holding the sensor device and walking along a designated track with specific postures. By considering the mobile user statistics reported in [28], three postures determining the degree of sensor tilt and its variation were applied to each experiment. The experimental environment is shown in Figure 9. The same experiment was conducted in a classroom (14.4 m × 8.4 m × 2.5 m) and a corridor (17 m × 2.1 m × 2.8 m). To cover a large measurement space, multiple LEDs were used in each experiment; however, positioning was performed based on the strongest single signal for each sample time. In the classroom, eight LEDs were placed between (−3.6 m, −1.2 m) and (3.6 m, 1.2 m) at width and length intervals of 1.2 m. Measurements were made by moving three times counterclockwise at an average speed of 0.4 m/s along the outskirts of a rectangular area from (−4.5 m, −2.1 m) to (4.5 m, 2.1 m) and 1.2 m high. Six LEDs were placed in the corridor between (−6.0 m, 0 m) and (6.0 m, 0 m) at intervals of 1.2 m. The measurements were made by moving three times clockwise at an average speed of 0.4 m/s along a rectangular area from (−7.5 m, −0.8 m) to (7.5 m, 0.8 m) and 1.5 m high.

**Figure 10:**
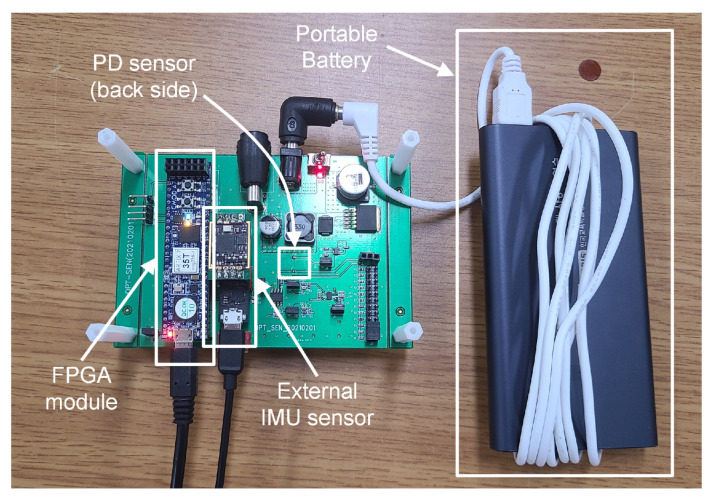
Receiver module (PD, IMU sensor).

In this study, we implemented transmission and receiving modules that could be controlled separately by an FPGA. The transmission part included an LED board with a white LED (SJ-3W-CW) and a main board transmitting a power source and an FPGA signal, allowing several LEDs to be driven. The receiving board included a silicon PIN photodiode (OSD5-E) and an amplifier circuit, receiving an optical signal from the FPGA through a 12-bit A/D converter operating at 12 MHz. The optical signals were modulated and demodulated by the orthogonality of the Hadamard matrix, and we applied the Manchester code to filter out the effect of the ambient light source. The inertial sensor data were measured using an external IMU sensor (EBIMU-9DOFV4). Data from both sensors were obtained at intervals of 10 Hz. In addition, the free movement of the device was implemented by a self-synchronization operation through an asynchronous signal sequence in a wireless environment. The power of the receiving board was supplied through a portable battery. The receiver module is shown in Figure 10.

Data collection was carried out while holding and moving the device at the height of the pedestrian’s chest, that is, 1.3 m above the ground, to maintain it evenly. In addition, walking scenarios according to the pedestrian’s posture were divided into three categories. Walking postures were classified as walking with the device held horizontally (Pose-1, vertical tilt of $-5^{\circ }$ to $+5^{\circ }$), walking while looking directly at the device (Pose-2, vertical tilt of ${+25}^{\circ }$ to ${+35}^{\circ }$), and walking while shaking the device freely (Pose-3, vertical tilt of ${-35}^{\circ }$ to ${+35}^{\circ }$). The horizontal tilt was kept as stationary as possible based on the walking direction in the first two postures, and was changed randomly in free movement.

**Figure 11:**
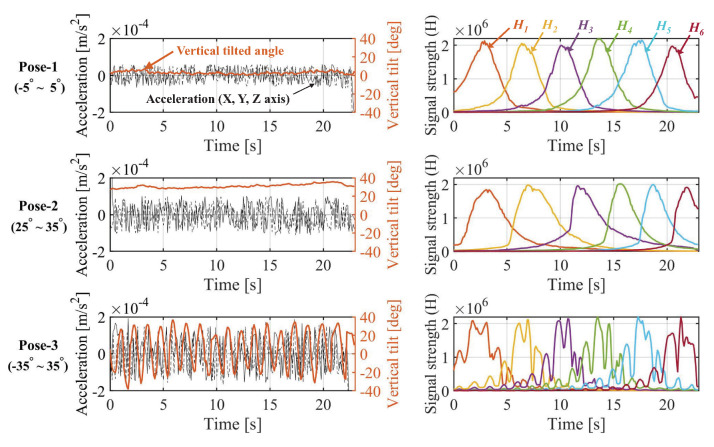
Part of the measurement data according to different postures.

Figure 11 shows the differences in the measurement data according to the three walking postures. The figure shows part of the data measured in the corridor. On the left side of the figure, the black solid line, dotted line, and dashed line indicate the acceleration data in each *X*-, *Y*-, and *Z*-axis converted to global coordinates, and the brown line represents the vertical tilt of the device. The right side of the figure shows the change in intensity of the optical signal received from each of the six LEDs over time. This figure shows that the noise of the inertial data is small when there is little change in posture, that is, the tilt of the sensor does not change significantly (Poses 1 and 2). When there is a large change in posture (Pose 3), the noise in the inertial data is large. However, when the posture changes slightly, the change in the received optical signal intensity is small. When the change in posture is large, the change in the signal intensity is large as well. In a posture with a specific tilt (Pose 2), the overall optical signal intensity appears to be sloped to one side according to the incident angle of the optical signal received by the PD.

**Figure 12:**
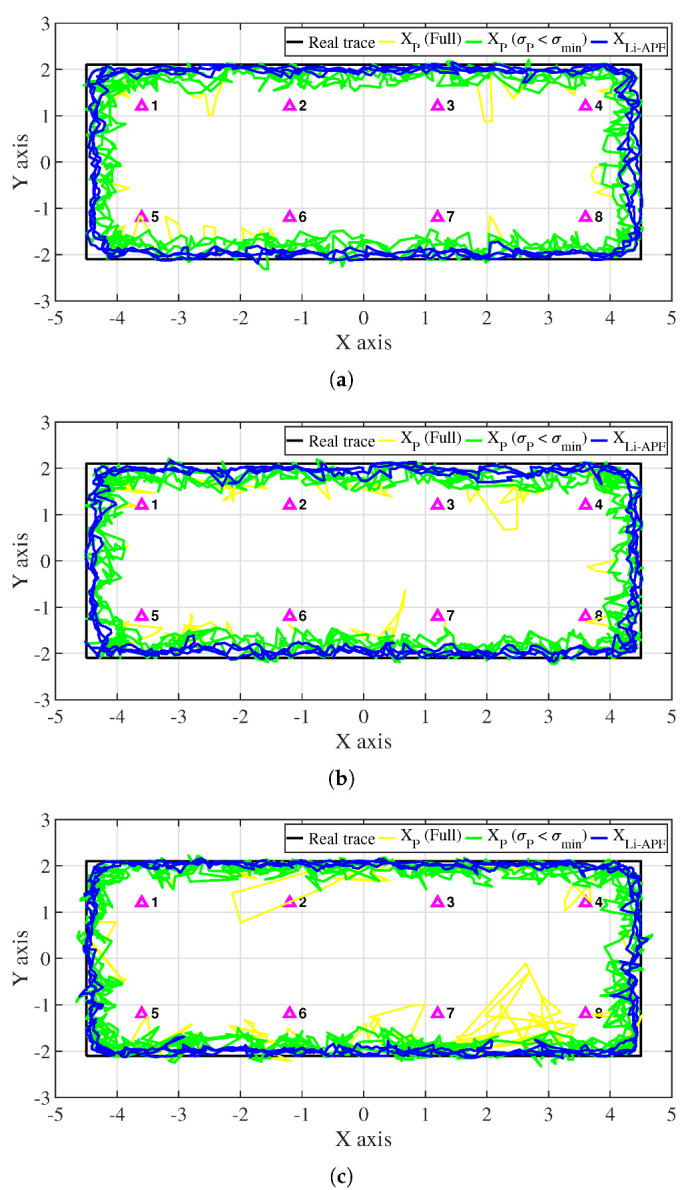
Experimental results in classroom environment: (**a**) Pose 1; (**b**) Pose 2; (**c**) Pose 3.

Figure 12 shows the results of the proposed algorithm in the classroom environment. The real trace made by connecting the four vertices is indicated by a black line. The yellow line represents the estimated position calculated during the particle update process, and the green line represents the position filtered according to the angle-weighted deviation condition ($\sigma_{P}\leq \sigma_{\min}$) among the results. The purple triangle indicates the location of a single LED. Here, the result removed by the reliability condition ($R_{P}<0.8R_{\min}$) is not included. The blue line represents the final estimated position of the Li-APF algorithm determined using the INS and particles. As in the measurement data analysis, the experimental results show that the results of particle positioning are formed close to the actual track without significant errors in Pose 3, where there are many changes in signal strength; Poses 1 and 2, where signal changes are relatively limited, show a slight increase in error in the particle results. On the other hand, particle results with a large error can be observed among the results for Pose 3 that do not meet the angle deviation condition; these are caused by a relatively large INS error and a measurement error occurring during optical signal reception.

The proposed method configures each particle using $P_{i}^{t}$, $P_{i}^{t-1}$ and the moving distance. The cost function and weight are then determined by the current and previous sensor measurements ($H^{t}$, $H^{t-1}$). Likewise, the next position of the particle ($P_{i}^{t+1}$) estimates the cost function and weight using $H^{t+1}$ and $H^{t}$, and can be resampled according to Equation (17). Here, if the change in measurements is insignificant ($H^{t-1}\approx H^{t}\approx H^{t+1}$), the scattered individual particles show flat cost functions and particle resampling does not occur. Conversely, when the measurement changes steeply, the number of particles satisfying continuity conditions is reduced, and there are more chances to eliminate inappropriate particles from the solution during the resampling process. In this regard, it can be expected that the cases with Pose 3 should show the fewest positioning errors in the experiments. On the other hand, the errors of the INS may be increased due to the rapid change in acceleration. In light of all of these considerations, we can examine the synthesized positioning results according to different poses.

**Figure 13:**
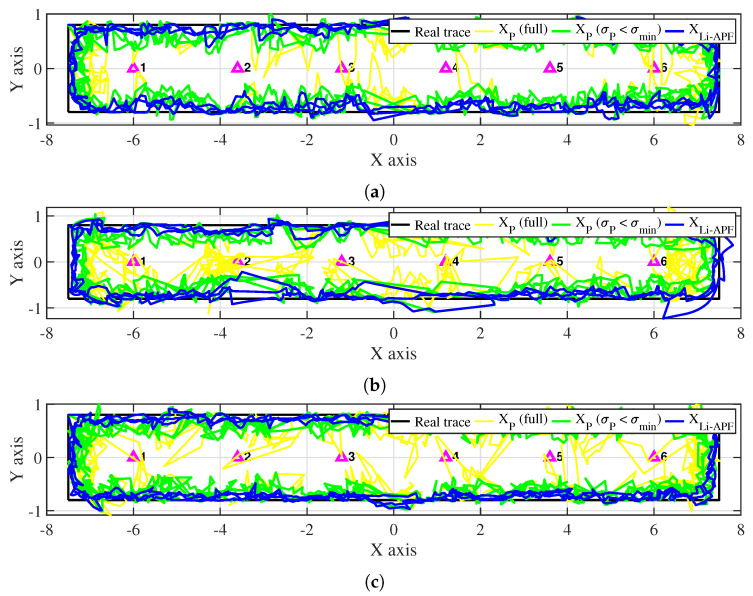
Experimental results in corridor environment: (**a**) Pose 1; (**b**) Pose 2; (**c**) Pose 3.

Figure 13 shows the tracking results in the corridor environment. Because the width of the space was relatively narrow, the distance between the LED and the trace was generally closer than in the classroom, and the corner was relatively short and changed direction quickly. Similar to the results in the classroom, the results with Pose 3 are stable, and the errors are relatively large in Poses 1 and 2. In addition, the results show that the deviation and positioning error of the particles are worse at the corner compared to the classroom.

**Figure 14:**
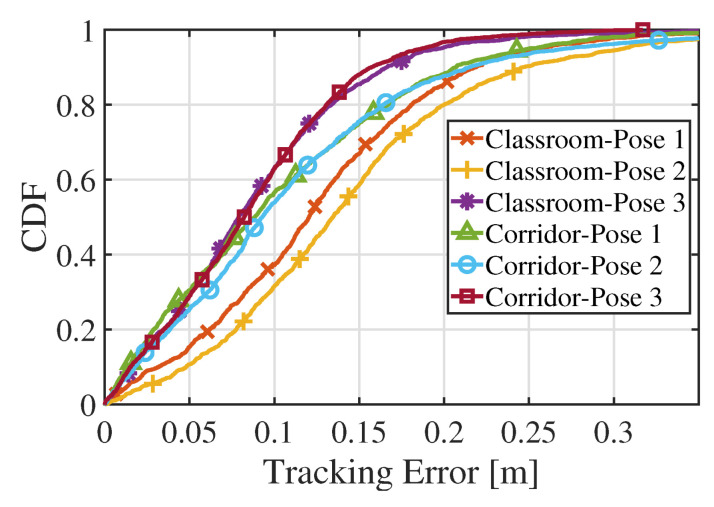
Tracking error CDF of Li-APF algorithm in each environment.

Table 2 summarizes the errors of the INS, particle positioning, and Li-APF in each experimental environment. In this table, INS refers to the result with general inertial navigation positioning that uses only the IMU sensor data. In the position tracking system, the path error is calculated by defining the shortest error distance in all directions from the real trace. The table reports that INS is an inappropriate system for long-term positioning due to cumulative errors, and that the proposed particle positioning has an average error of 21.63 cm. Finally, the proposed Li-APF algorithm is reported to have excellent overall performance, with an average path error of 10.89 cm. Figure 14 shows the cumulative distribution function (CDF) of the Li-APF path error for each experiment. As previously observed, the error with Pose 3 is smallest in each place, and the result with Pose 1 with a lower INS error was better than that of Pose 2. In addition, in all cases it can be seen that the top 80% of results have an error within 10–20 cm.

## 6. Conclusions

In this study, a single LED, single PD-based sensor tracking algorithm was proposed with a novel light-based adaptive Bayesian tracking method. The proposed method effectively solved the structural limitations of the general VLP system by requiring only one signal pair and considering the tilt of the receiver. As the Li-APF borrows the concept of particle filters to effectively reduce the computational burden, the proposed system is robust for nonlinear systems with non-Gaussian noise distribution while being less burdensome on online tracking systems. The algorithm proposed in the was reviewed in depth via simulation. In addition, an experiment was conducted according to three device handling scenarios, moving a total of 79.2 m and 99.6 m in a classroom and corridor, respectively; the average error was found to be 10.891 cm. With the proposed method it is expected that real-life applications of VLP systems can be realized, with great potential in the development of various application fields.

## Figures and Tables

**Table 1:** Parameters of the proposed algorithm.

Symbol	Parameter
$H_{l}^{t-1},H_{l}^{t}$	The intensity of the received optical signal of the *l*-th channel previous and current.
$H^{t-1},H^{t}$	The reference (strongest) received optical signal strength previous and current.
$V_{\mathrm{INS}},S_{\mathrm{INS}}$	The instantaneous speed and unit time movement of the sensor.
θ,β	Vertical and horizontal tilt of the device.
$H^{\prime }\left(\cdot \right)$	An ideal signal strength calculated according to a parameter.
$H^{0},P^{0},T^{0}$	The initial reference optical signal strength, position and tilt of the *i*-th particle.
$W_{\mathrm{init}}$	Initialization value of particle weight.
$P_{i}^{t-1},P_{i}^{t}$	The position of the *i*-th particle previous and current.
$T^{t-1},T^{t}$	The tilt of the particles previous and current.
$C_{i}$	The cost function value of the *i*-th particle.
$W_{i}^{t-1},W_{i}^{t}$	Weights of the *i*-th particles previous and current.
$W_{i}^{t*}$	Normalized current weight.
$R_{P}^{t-1},R_{P}^{t}$	Reliability of particles previous and current.
$\sigma_{P}$	Weighted deviation of the particle angles.
$P_{O}^{t-1},P_{O}^{t}$	The virtual position of the previous and current device.
$P_{\mathrm{typ}}^{t-1},P_{\mathrm{typ}}^{t},P_{\min}^{t-1},P_{\min}^{t}$	The virtual reference position of the previous and current device.
$\Delta_{\mathrm{typ}}^{t-1},\Delta_{\mathrm{typ}}^{t},\Delta_{\mathrm{typ}}$	The virtual position errors and generalization values of the previous
$\Delta_{\min}^{t-1},\Delta_{\min}^{t},\Delta_{\min}$	and current reference device.
$C_{\mathrm{typ}},C_{\min}$	The reference cost function value calculated through the virtual device position.
$R_{\mathrm{typ}},R_{\min}$	The reference reliability calculated through the reference cost function value.
$\sigma_{\min}$	The reference minimum weighted deviation of the particle angles.
$N_{P}$	Total number of particles.
$X_{P}^{t-1},X_{P}^{t}$	The results of the particle positioning previous and current.
$X_{\mathrm{INS}}^{t-1},X_{\mathrm{INS}}^{t}$	The result of the INS positioning previous and current.
$X_{\mathrm{Li}-\mathrm{APF}}^{t-1},X_{\mathrm{Li}-\mathrm{APF}}^{t}$	The result of the Li-APF positioning previous and current.

**Table 2:** The results of the tracking experiment in various environments.

Environment		Average Error [cm]		
(Place/Tilt)		INS	Particle	Li-APF
Classroom	Pose-1 ($\;-5\;∼\;5^{\circ }$)	122.526	25.454	12.515
	Pose-2 ($\;\;25\;∼\;35^{\circ }$)	365.746	27.578	14.367
	Pose-3 ($-35\;∼\;35^{\circ }$)	527.970	18.141	8.569
Corridor	Pose-1 ($\;-5\;∼\;5^{\circ }$)	153.775	19.210	10.222
	Pose-2 ($\;\;25\;∼\;35^{\circ }$)	203.258	22.890	11.014
	Pose-3 ($-35\;∼\;35^{\circ }$)	607.655	17.332	8.658
Average		330.155	21.634	10.891

## Data Availability

Not applicable.
